# Developing the Art–Technology Intergenerational Community Program for Older Adults' Health and Social Connectedness

**DOI:** 10.3389/fpubh.2021.589589

**Published:** 2021-06-23

**Authors:** Jinsil Hwaryoung Seo, Annie Sungkajun, Brittany Garcia

**Affiliations:** ^1^Soft Interaction Lab, Department of Visualization, Texas A&M University, College Station, TX, United States; ^2^Creative Technologies/Graphic Design Department, Illinois State University, Normal, IL, United States

**Keywords:** older adults, art, health, well-being, social connectedness

## Abstract

As the older adult population increases, research investigating how to support their health and well-being has become more urgent. This paper discusses the development of the art–technology intergenerational community (ATIC) program for older adults in Bryan and College Station, Texas. The program's purpose was to help improve older adult's health, well-being, and social connectedness. During the program, participants attended four sessions across 4 weeks, creating interactive art projects such as light-up cards, pop-up cards with light, interactive light painting, and interactive soft circuit ornaments. Preliminary studies allowed researchers to refine making materials by designing easy-to-follow fabricated circuit templates. Participants were able to create interactive art by using various materials such as light-emitting diodes (LEDs), copper tape, coin-cell batteries, and conductive thread. A total of 18 participants aged 60–83 participated in the ATIC program. Participants were asked to complete pre- and post-study questionnaires which assessed older adults' subjective health or well-being, feelings of intergenerational connectedness, and attitude about art and technology. Video data were captured for qualitative analysis on the art creation process, cognitive health, and social connectedness of the participants. Our findings show that those who participated in the ATIC program had improved perceptions of their own health and intergenerational relationships. There were also significant differences between pre- and post-study conditions for positive and negative affect. Qualitative results showed that the program participants were engaged in the art-making process and that creations helped to support intergenerational relationships with the student volunteers as well as their own family members.

## Introduction

The health and well-being of older adults have become more important because the older adult population is growing rapidly. In 2016, 49 million people, 14.5% of the US population, were aged 65 or older, and the number is projected to climb to about 98 million (25%) by 2060 (1). As a result, the United States faces growing challenges and issues in terms of older adults' healthy aging. Much effort on research and many practical services were put in place to support that (2).

One of the collective efforts is providing art-based older adult programs. Research has shown that creative practices including dance, creative writing, music, theater, and visual arts have positive impacts on improving health, well-being, and independence of older adults (3–9). Participating in community art programs is an effective way of promoting social interaction and psychological well-being (10, 11). Creative activities for older adults have been considered to be relatively low cost and can be made easily available to seniors throughout many local facilities (12).

Most recently, interactive art and crafts, making, and coding have been introduced to the older adult population (9, 13–15). These activities contribute toward the interactive experience with artwork. In addition, there is a growing number of artists and researchers who have focused on the educational relevance of these activities as well as health-related potentials (16–18). Interactive art is a form of art that involves various levels of participant's sensory engagement with the artwork (7, 19). Some interactive arts suggest that participants' active input to finish the works allows them to become part of the artwork themselves, which in turn may stimulate older adults' cognitive functions. Coding and making activities have been popular in younger community groups (i.e., under 25). These making activities involve learning interactive concepts, figuring out functionalities, and implementing art forms that can have a significant impact on how people think and feel. These activities allow creative expression through an algorithmic thinking process (20). Through the coding and making processes, we can help older adults to set a “can-do” mindset (20) that encourages them to act and to take control of their lives.

Healthy aging is not an individual older adult's problem to take care of; it requires family and community-based support. Families provide foundational affective bonds and share responsibilities, such as caregiving and intergenerational transfers of knowledge and wealth. Research indicates that there are positive correlations between intergenerational or familial support and subjective well-being and mental health among older adults (21–23). However, more than one-third of adults aged 45 and older feel lonely, and nearly one-fourth of adults aged 65 and older are considered to be socially isolated in the United States (24). The risk of social isolation and loneliness has increased because it is closely related to living alone, the loss of family or friends, chronic illness, and hearing loss (25, 26). Therefore, a community-based effort for supporting intergenerational relationships and social interaction is critical.

We drew inspiration from the potential opportunities of interactive art and craft practice and started a small art workshop in the cities of Bryan and College Station, Texas. As more older adults showed interests and participated in our events, our program became a regular weekly program at multiple locations. In the early workshops, we introduced interactive art and craft technology to participating older adults in local assisted living and nursing homes. Participants integrated a paper-based electronic circuit into various art forms (e.g., painting, drawing, and sewing) and created interactive art. From preliminary studies, we learned that such programs could hold great potential to engage older adults and significantly improve their daily lives (9, 27). We also learned that technical support for this kind of program is critical because if materials are not accessible, participants will not produce meaningful experiences. Therefore, we adjusted materials and developed paper templates focusing on usability. In addition, we actively invited college students to assist and work with older adults in our workshops. This way, participants were able to overcome technical barriers and created what they wanted with the assistance of the students. This became a framework of our program and led to the present study. This article examines how participating in the art–technology intergenerational community (ATIC) program may impact an individual older adult's health, well-being, and social connectedness.

## Background

We find the foundation of our practice from Erikson's psychosocial human development theory and continuity theory. Art activities are known for supporting older adults who have difficulties expressing themselves and can enhance individuation, which is important to well-being in older adults (28). According to art therapists, supporting and encouraging making art is effective when working with older adults. Studies with older adults and art have centered around dementia, restricted ability to communicate, and lifelong mental illness. Art therapy is most effective when older adults understand what is considered healthy and adaptive during the life stage they are in (29). Cohen et al. (30) conducted a study with older adults in art programs investigating emotional and health benefits; results showed that participants improved both emotionally and physically. Erikson's theory of psychosocial human development addresses adult development, specifically the need to seek ego integrity. In each developmental stage, a person has conflict that they must overcome to move to the next stage. The final stage is when individuals realize that their life has had meaning despite failures, and they feel a need to share this wisdom with others. Through this, the older individual will feel a sense of connection with younger generations. Chapin Stephenson stated that by participating in art activities, older adults stay involved, connected, and exhilarated (29).

Like Erikson's psychosocial human development theory, continuity theory focuses on an individual's ability to link things from the past to changes in their future. With regard to older adults, the premise of continuity theory is that adaptation to change is done by using strategies to maintain continuity in their lives, both internal and external. Internal continuity refers to the forming of personal links between new experiences and previous ones, and external continuity refers to interacting with familiar people and living in familiar environments (31). Art allows older adults to maintain continuity by providing a visual link for them to explore past and present experiences (32). These theories guide us to develop the ATIC program that focuses on participants' perspective health and connecting with younger generation through art activities.

## Material: The Atic Program

The ATIC program consists of four sessions over the span of 4 weeks, with each session taking 1 h. The program focuses on encouraging participants to engage in art and technology creations with undergraduate volunteers. Each week, participants worked on a new project, gaining an understanding on how interactive techniques can be utilized in their art. The workshop includes four activities: *light-up cards, pop-up cards with light, interactive light painting*, and *interactive soft circuit ornaments*. The activities incorporated basic forms of art, such as painting, drawing, paper folding, and sewing. In the workshop, participants work very closely with undergraduate volunteers who work as assistants and collaborators in each session. Workshop participants shared their hobbies, moments in their lives, and personal interests.

### Custom Materials Development

**Figure 1:**
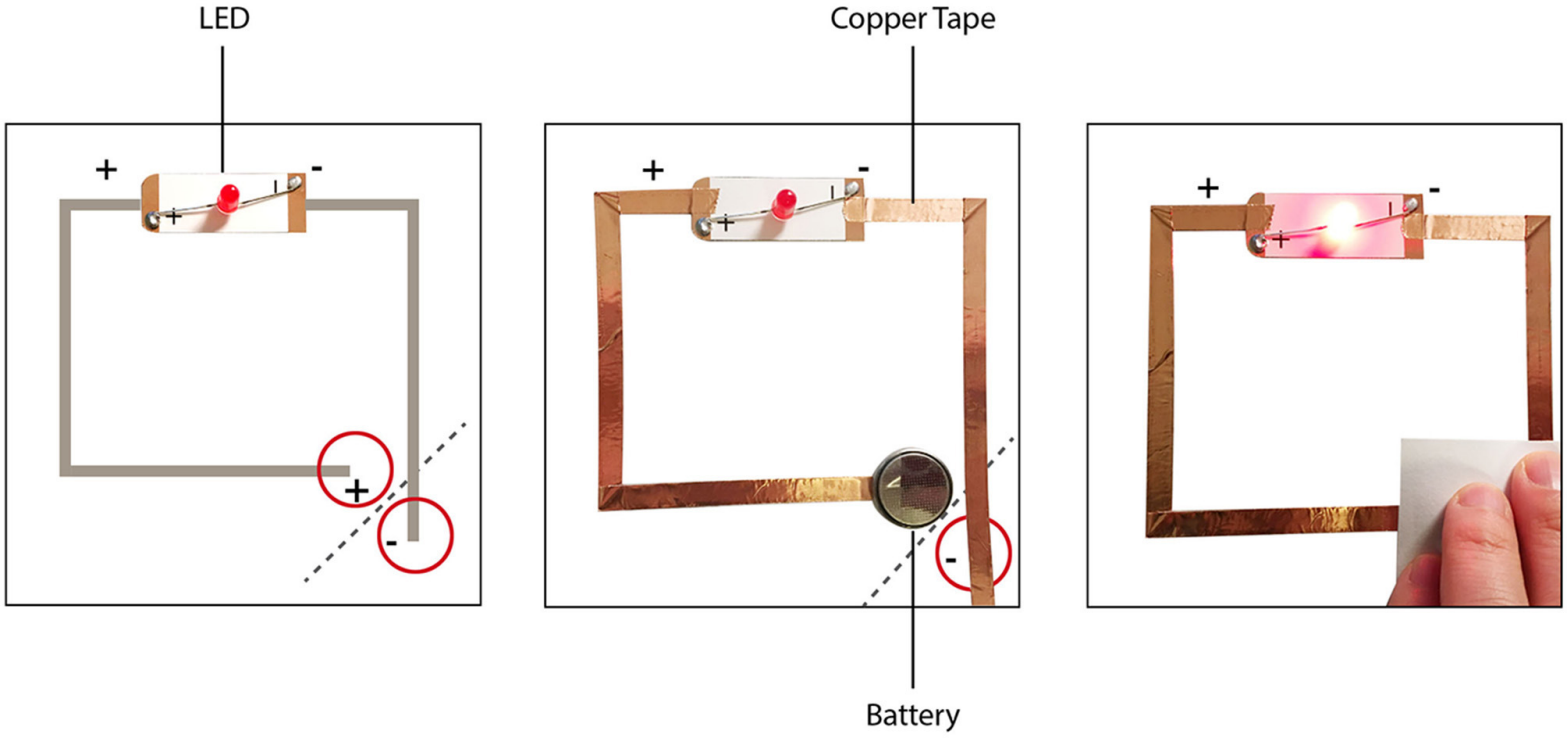
Paper circuit template.

Through previous studies conducted, we learned how difficult it was for older adults to handle small electronics, as well as hesitant to handle them. Despite the parts being very simple and not enough to cause harm, the question “Will I get shocked?” was still commonplace. In order not only to make these parts accessible but also to make the participants feel comfortable, we chose to fabricate our own paper circuit template for light-emitting diodes (LEDs) using cardstock paper (Figure 1).

These templates allowed participants to know where to place the LEDs, based on their position, and also helped to match the positive and negative sides so as to avoid error. Lines indicated where to place the copper tape, while a circle helped to show where to place the coin-cell batteries provided. On the corners of the templates, a dashed line showed participants where to fold, in order to create the interaction, creating a connection point between the copper tapes to the battery (Figure 1).

### Four Curated Activity Sessions

#### Activity 1: Light-Up Cards

Materials: LEDs, paper, copper tape, coin-cell battery

**Figure 2:**
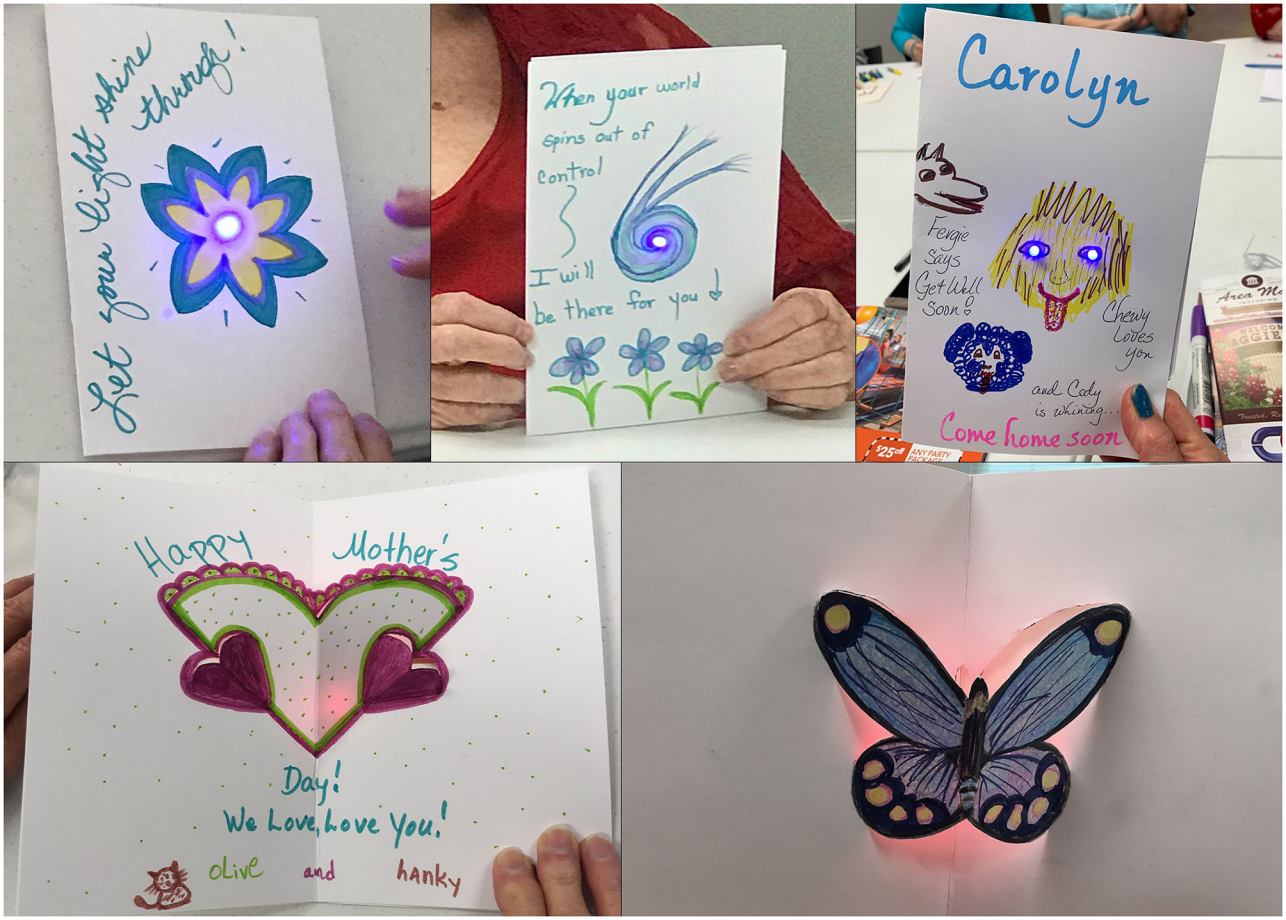
Light-up cards and pop-up cards created by participants.

After participants filled out the pre-study questionnaire, we introduced creating light-up cards. A simple fold connection was utilized so that it could act as a “switch” to turn the light on, while also preserving the battery power. The circuitry portion of the cards was then placed into folded pieces of paper, so that the light would be able to shine through and allow them to create their art, while incorporating the light into their designs. Due to the card's free form, participants were creative with their design choices, ranging from utilizing the lights as noses for drawn dogs or to light up a drawn emergency vehicle in a get well soon card for a family member (Figure 2). At the end of the session, participants presented their works and commented on each other's card.

#### Activity 2: Pop-Up Cards With Light

Materials: LEDs, paper, copper tape, coin-cell battery

For the second week of our program, we chose to continue a similar circuit procedure, incorporating the art of kirigami (the cutting and folding of paper) in order to create pop-up designs. Simple pop-up templates, such as circles and butterflies, were provided to give them a base for their design. However, they were also free to create their own and explore other options after they finished a template. Participants had to make decisions on how to incorporate their chosen color of light into their design. The light within the pop-up card acted as a backlit glow for their designs. Most of the participants remembered the use of copper tape and which way the LED and battery should face. The template designs (butterflies, hearts, and a simple circle) guided participants' final pop-up cards. The butterfly design allowed them to either reference real butterfly wing designs or to create their own. The heart design was often used to show sentiments of love for family and friends (Figure 2).

#### Activity 3: Interactive Light Painting

Materials: LEDs, watercolor paint, copper tape, coin-cell battery, paper

**Figure 3:**
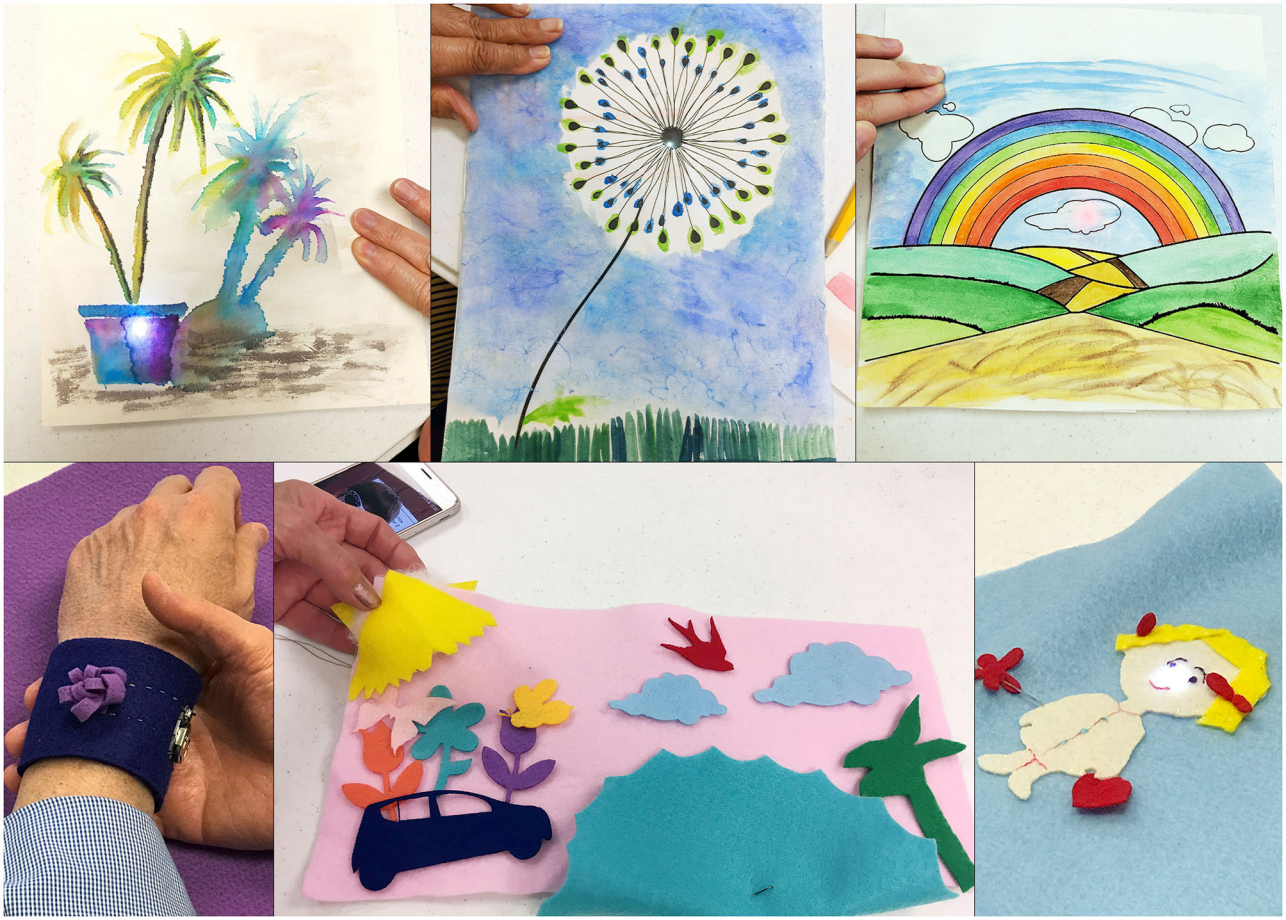
Light-up paintings and soft ornaments created by participants.

The third activity was to create watercolor paintings that incorporated the same simple light circuits they have learned. Participants were welcome to be experimental with their circuitry or use pre-made circuit diagrams with sample designs they could paint. Sample designs included dandelions to rainbows. A live demonstration was shown to explain the process of water coloring to those who had no prior experience. Participants who were experienced painters helped and gave advice to beginners, showing unique techniques such as “watercolor splattering,” a process to create splashes of dotted color randomly across the page. Most participants experimented with painting certain areas, whereas some chose to begin with the background. Figure 3 includes some light-up paintings done by the workshop participants.

#### Activity 4: Interactive Soft Circuit Ornament

Materials: Felt, conductive thread, thread, coin-cell battery, LEDs, battery pack

In our last activity, we introduced the participants to a sewing project where they created soft, light-up objects out of felt. In the prior activities, they used copper tape as connection points between their LEDs and the battery. For this soft circuit, we introduced conductive threads in order to sew the connections between the LEDs and the provided battery packs. The materials provided for this activity were pre-cut fabric objects, using a laser cutter, that included shapes such as flowers, birds, a child, dogs, and cats. We also provided base shapes such as rectangles and circles that allowed participants to be creative for the usability of the artwork. Some saw that the rectangular-shaped bases could be effective as light-up bookmarks, while another participant thought to create a wristband for their daughter. Most participants used the light to illuminate specific objects they had sewn into their base, such as flowers, or in order to illustrate a scene that they had created (Figure 3).

## Methods

The goal of our work is to understand how community activities involving art and technology with older adults and undergraduate students could improve older adults' subjective health or well-being and social connectedness.

### Study Description

Our study consisted of a pre-study questionnaire, four workshop sessions (ATIC program), and a post-study questionnaire. The workshops were held at two locations: the Art Council of Brazos Valley and Southwood Community Center in College Station, Texas. Each workshop session was an hour long and consisted of a short briefing on the art topic, a Q&A period, and then the art activity itself. Data from these sessions were gathered via video recording and questionnaire. Each session was recorded so that dialogue and actions could be coded objectively. Questionnaires were given out twice, once at the very beginning of the study and once at the very end of the 4 weeks. The intent for these types of data collection was to record the baseline, the process, and the outcome. The questionnaire includes questions about older adults' subjective health or well-being, their feeling of intergenerational connectedness, and their attitude about art and technology. Questions were collected from standard measures including self-reported health, positive and negative affect, art and technology interest, self-efficacy, and intergenerational relationships. We composed several standard questions using associate scales (33–36).

### Program Participants

We recruited older adults, aged 60 and up, who had not been diagnosed with any age-related mental illnesses and who lived independently and did not require any form of third-party caregiver. Recruitments were conducted through the Texas A&M mailing list for retired faculty and staff. In addition, participants were able to register for the program at the workshop venues. Because the ATIC program requires a small group setting to provide individual support and active participation, we recruited 8–10 participants per workshop. Eighteen participants (15 females and three males, aged 60–83) who finished at least three sessions were included in the study data.

In the ATIC program, undergraduate volunteers took critical roles as a workshop assistant, team member, collaborator, and younger friend. They particularly supported one-on-one communication and social engagement. Nine students participated in the program: four students from the art department and five students from different departments (psychology, biomedical science, and chemistry). All of them were interested in either working with older adults or making pieces of art. Some students utilized this opportunity for developing social interaction skills for their future career.

### Data Collection and Analysis

We collected both quantitative and qualitative data to measure how art and technology activities could improve participants' subjective health or well-being and social connectedness. The questionnaire included questions from standardized psychological measures for self-reported health, mood, perceived control, functional health, well-being, art interest, and technology self-efficacy. We compared pre- and post-responses on measures at time 1 (before the intervention) to time 2 (after the intervention).

We utilized a self-reported health measure to assess perceptions of current health. Participants responded to questions such as “How would you rate your health at the present time” and “How much do health problems limit your daily activities”; questions were scaled on a 5-point Likert scale. To assess older adults' sense of control, participants responded to three statements taken from a standard six-item measure of confidence (34). Participants were asked on a scale of 1 (strongly agree) to 5 (strongly disagree) to indicate their level of agreement with statements such as “I can do just about anything I set my mind to,” “If I want something, I go out and get it,” and “I am a go-getter.” To assess older adults' sense of overall well-being, participants completed the Subjective Well-being Scale [SWLS; (35)]. The scale consists of five items examining how satisfied the individual is with his or her life, with response options on a 7-point scale (ranging from strongly disagree to strongly agree), for a total positive score of 30. Older adults' interest in art was assessed by using a single-item question (e.g., “How likely would you be to engage in other art/technology workshops”) to be answered on a Likert scale from 1 (very unlikely) to 5 (very likely). Older adults' confidence in their ability to use technology was assessed by using a single-item question (e.g., “I am confident with my ability to learn and use technology”) to be answered on a Likert scale from 1 (strongly disagree) to 5 (strongly agree). To assess older adults' sense of intergenerational connectedness, participants completed a questionnaire from the Family Exchange Study (36) designed to assess support given, support received, and family support beliefs.

Finally, to assess current mood, participants completed a measure of positive and negative affect [Positive and Negative Affect Schedule (PANAS); (33)], in which participants were asked to rate the extent to which 20 different adjectives (10 positive and 10 negative) describe how they are feeling at the current time, using a 5-point scale (1 = not at all; 5 = extremely). The positive affect words were *interested, cheerful, energetic, excited, inspired, strong, confident, loved*, and *enthusiastic*, and the negative affect words were *distressed, incapable, miserable, nervous, scared, hostile, dissatisfied, irritable, pathetic*, and *afraid*. For analysis, pre- and post- study questionnaire data were entered into a spreadsheet, and a statistical analysis program, SPSS, was used to run paired-sample *t*-tests. The positive and negative affect words were scored and averaged from participant scores for the words.

The qualitative data were generated by asking pre-prepared open-ended questions during the workshop sessions. A total of 17 h of video recordings were collected. The videos were coded using the MAXQDA qualitative analysis application. A team of researchers conducted open coding as a first step for video analysis by focusing on individual participant speaker turns with other participants. A speaker turn was defined as consisting of a discourse excerpt (one or more conversational turns). The team met again and discussed arising categories such as the art process, participant health or well-being, and social connectedness. Researchers had multiple rounds of meetings to determine a proper coding scheme. From these discussions, researchers agreed upon three overarching codes: the *art creation process* was to note the process each participant went through when creating their art, *cognitive health* was to analyze the participants' mental well-being, and *social connectedness* was the moments where participants were making social connections to other participants and researchers.

## Results and Discussion

### Participants' Backgrounds and Interests

This paper discusses the ATIC program for older adults; the purpose is to help improve older adult's health, well-being, and social connectedness. For this study, previous art experience was not required, but 12 participants had art-related hobbies including painting, sewing, crafting, silk screening, weaving, gardening, and jewelry making. There were six participants who did not have much experience with being artistic, but they wanted to try in the workshop. However, these participants still had the mindset that they were not good at making art. They often said, “I am bad at painting” (S19-06) and “I'm a horrible drawer” (S19-19).

Many participants decided to participate in the ATIC program to make art and meet other people. Participant *S19-03* commented, “I just want to take some art and meet some people too!” Similar notions were shared by *S19-02*, “I wanted to try something new.” Some also participated in the workshop to connect with their grandchildren who study art, such as S19-28.

### Quantitative Results

Paired-sample *t*-tests were conducted using SPSS to analyze the data collected, which can be found in Table 1.

**Table 1:** Quantitative *t*-test results.

		**Mean**	**SD**	***t***	***df***	***p***
Subjective health	Pre	4.08	0.73	−4.014	17	0.001
	Post	4.56	0.59			
Self-efficacy	Pre	5.84	0.80	−2.22	17	0.04
	Post	6.04	0.69			
Positive affect	Pre	3.83	0.65	−2.70	18	0.015
	Post	4.03	0.67			
Negative affect	Pre	1.19	0.24	2.695	17	0.015
	Post	1.08	0.176			
Intergenerational	Pre	5.78	1.00	−2.72	17	0.015
connectedness	Post	6.17	0.71			

### Qualitative Results

From the qualitative analysis process, we categorized codes into three areas: *art creation process, cognitive health*, and *social connectedness*. The following presents themes that emerged from the workshop programs' video data.

#### Art Creation Process

The workshop participants were very active in the process of art making. They followed the instructions very well and supported each other by providing feedback, helping in design choices, and sharing materials. Participants also supported each other to make more interesting or polished works.

##### Making Artworks for Loved Ones

Many participants used the workshop as an opportunity to create something for their loved ones: family members and close friends. In the process of making, participants often shared stories about the person who they were making the art for to others in the workshop.

“Today is my daughter's birthday. She has two cats. So I made a card from the cats. She will love it.” (S19-20)

“My friend got a big surgery recently. I want to make a card for her.” (S19-23)

##### Providing Feedback on Experimentation and Iteration

The art creation processes became experimental and iterative. Participants tried different colors and materials, seeking other participants' and student volunteers' opinions about their artwork. Participants started with suggesting colors and simple ideas while actively participating in brainstorming ideas and finding complex solutions for electronic circuits with other participants and student volunteers.

“Where are you going to put the light” (S03) “I thought it was gonna like go in the middle and light up the whole thing but … I could put the light under like that tree? Like right there. I don't know, maybe. I don't know, I don't think it matters.” (S19-01) “You can always move it around and then tape it down where you like it.” (S03) “Oh okay!” (S19-01)

“I'm just not seeing the butterflies maybe we just need to come up with something else.” (S19-14) “Butterflies will be great. What about this design?” (S05) “Oh, I like that.” (S19-14)

##### Sharing Personal Knowledge and Skills

Some experienced participants actively helped other participants who had never done any art projects before. During the painting and sewing sessions, skilled participants were able to display their knowledge and skills, which were transferred among the group.

“So the trick about working with watercolor is having a blow dryer because you really need to get it dry before you move on. This is not my first time” (S19-33)

“Oh I lost my thread.” (S19-07) “You can tie a thread about the needle.” (S19-03)

#### Cognitive Health

The workshop program created positive impacts on participants' cognitive health. Even though all our participants were healthy and independently living, their participation in the art program made them more active and engaged with various cognitive processes.

##### Creativity

The workshop program was designed to evoke creativity in older adults. Participants thought technology-based art making was very thought provoking and unique. Their projects started from examples and templates, but participants integrated their personal stories and experiences into the projects.

“You know, I never thought about putting those kinds of things together. That's just, wow! That's interesting.” (S19-07)

##### Keeping Their Mind Active

The electronic circuit component made participants intellectually engaged. To create their work, participants had to remember the positive and negative sides of an LED and move their circuit around their works (i.e., painting, card, and soft ornament). This process could be cognitively demanding and improve one's thinking ability.

“What I'm gonna do is I'm gonna put my light here and I'm going to put my positive side right there and I'm gonna run my thread right there.” (S19-23)

“Can I make this side negative? How can I connect it to negative? … Okay that just made my life a bit easier.” (S19-33)

##### Excitement and Happiness

Maintaining a good mood enhances cognitive function in older adults. Happiness and mood could support the quality of life of older adults (37). Throughout the workshop program, there were lots of laughs and many excitements expressed by the participants and student volunteers.

“That's what I miss about work is all the laughter you know? We just laugh so much.” (S19-31)

“Hah! My light works!” (S19-05) “Oh it looks good! I like it.” (S19-08)

#### Social Connectedness

**Figure 4:**
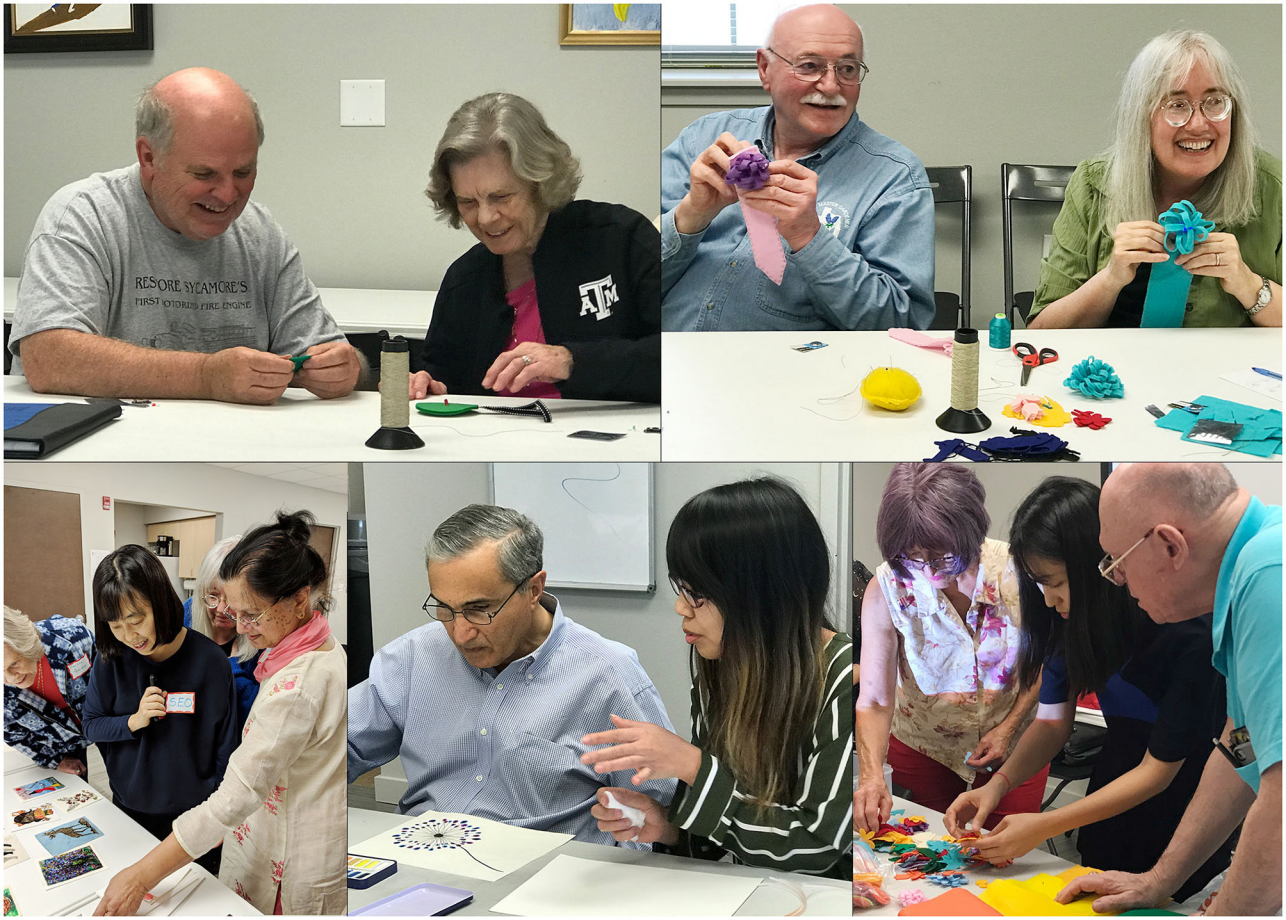
Participants working collaboratively in the ATIC program.

Participating in social activities has been suggested to lower the risk of some health problems and improve well-being (38). In the ATIC program, participants were connected with other older adults and volunteer students (Figure 4).

##### Sharing Personal Stories

Through multiple sessions of the workshop, participants became close and shared personal stories about themselves, family members, and friends with other participants, researchers, and student volunteers.

“My hobbies are embroidery, I recently won a contest by embroidering a denim jacket.” (S19-01)

“Today I have to leave early because I have to go to my friend's wedding. He is 92 years old. This is a special wedding for a 92 year old man.” (S19-02)

##### Planning Beyond the Workshop

We found that while participants worked together, developing social networks continued after the workshop.

“We're gonna have to find some other class we can take together” (S19-21) “No kidding y'all I was just thinking that. I would like to get all y'all's emails.” (S19-24) “A family reunion, a class reunion.” (S19-24).

##### Intergenerational Relationship

Workshop participants reported that they usually communicate with their children or grandchildren via text message, FaceTime, or Skype. They sometimes send them birthday cards. In the beginning of the workshop, their perception about new technology around younger generations was negative, because they thought technology did not support more face-to-face relationships in society.

The workshop program led participants to reconnect with their children or grandchildren via their art creations.

“I have a granddaughter that's 12 and she just loves to make her own cards and she does very complicated and they're folded. I would like to show her what I made here.” (S19-14)

Something unique about this program is that undergraduate students participated in the workshop with older adults. Student volunteers worked as workshop assistants as well as collaborators. In the beginning of the workshop, they talked only about workshop projects and materials.

“Would you help me find images of butterflies?” (S19-02)

“How can we draw an ambulance?” (S19-05)

In the later sessions, the workshop became an intergenerational art club to share many things: university life, career, job search, favorite movies, favorite books, hobbies, and so on.

## Discussion and Conclusion

We created an intergenerational program for older adults that involves art- and technology-based activities, called ATIC. In the ATIC program, older adults created personalized greeting cards, pop-up cards, paintings, and soft ornaments using custom-designed paper-circuit templates. This program was supported by college student volunteers who worked as workshop assistants and collaborators. Quantitative results revealed that there were significant differences in the scores (subjective health, self-efficacy, and participants' perception about the younger generation) between pre- and post-study conditions. When comparing pre- and post-study results for subjective health, they showed an increase; these results coincide with the results of those participating in art therapy and art education in that improved cognition is observed in older adults by providing a dynamic learning opportunity for them to engage their cognitive motor and social abilities (39). Results from pre- and post-study self-efficacy scores also show significant differences as well; therefore, by participating in the ATIC program, older adults' self-efficacy increased. This is similar to other studies investigating older adults and self-efficacy while attending art therapy or art workshops (39). Positive affect showed significant pre- and post-study results; however, the scores for negative affect significantly decreased. These two findings support the notion that by participating in the ATIC program, older adults have the possibility to improve positive affect while decreasing negative affect (40). In terms of the participants' perception about the younger generation, their views also improved, meaning that the older adults' views on the younger generation went from a negative outlook to positive. By working with undergraduate volunteers, the older adults were able to share experiences while creating art. This kind of stimulation, which is in line with Erikson's theory (29), allows older adults to reflect on their past and share these memories with the undergraduate workers, in turn causing their views of different generations to change.

These findings were also supported by qualitative results. The participants were very engaged in the ATIC art-making process. Their creations reflected their love to strengthen existing relationships with younger generations. Participants became more connected to their children and grandchildren, extended family, and close friends and expanded their relationships to the student volunteers. The workshop participants shared their knowledge and life experiences with volunteer students and others, creating a support system among the participants. Participants utilized various art supplies that were provided, which helped to enhance their ability for creativity. Findings such as these are common among other art studies such as Cantu and Fleuriet (41), which showed that art creation in art programs can promote well-being.

Overall, the ATIC program was effective in improving subjective health and social/intergenerational connectedness in older adults. This program could be modularized and disseminated to other community facilities including art centers, senior community centers, and assisted living homes to support older adults' health and social connectedness. After the study was ended, our team shared all workshop materials on our website (http://softinteraction.com/) and continued to support local community programs. Activity directors at local programs for older adults have access to them and can integrate the program into their activity programs. Our team will make sure materials are updated and be easily accessible by program directors. We will also visit the local community center once a month and invite student volunteers to engage with older adults.

## Data Availability Statement

The raw data supporting the conclusions of this article will be made available by the authors, without undue reservation.

## Ethics Statement

The studies involving human participants were reviewed and approved by Texas A&M University. The patients/participants provided their written informed consent to participate in this study. Written informed consent was obtained from the individual(s) for the publication of any potentially identifiable images or data included in this article.

## Author Contributions

JS is the PI in this research and contributed to design/run the workshop program, data collection, data analysis, and write the final report. AS contributed to prepare/fabricate workshop materials and run the workshop, and write the final report. BG contributed for quantitative and qualitative data analyses and editing the final report. All authors contributed to the article and approved the submitted version.

## Conflict of Interest

The authors declare that the research was conducted in the absence of any commercial or financial relationships that could be construed as a potential conflict of interest.
